# A 16-year prospective cohort study to evaluate effects of long-term fluctuations in obesity indices of prediabetics on the incidence of future diabetes

**DOI:** 10.1038/s41598-021-91229-9

**Published:** 2021-06-02

**Authors:** Shahla Safari, Maryam Abdoli, Masoud Amini, Ashraf Aminorroaya, Awat Feizi

**Affiliations:** 1 Isfahan Endocrine and Metabolism Research Center, Isfahan University of Medical Sciences, Isfahan, Iran; 2 Department of Biostatistics and Epidemiology, School of Health, Isfahan University of Medical Sciences, Isfahan, Iran

**Keywords:** Endocrinology, Health care, Epidemiology

## Abstract

This study aimed to evaluate the patterns of changes in obesity indices over time in prediabetic subjects and to classify these subjects as either having a low, moderate, and high risk for developing diabetes in the future. This study was conducted among 1228 prediabetics. The patterns of changes in obesity indices based on three measurements including first, mean values during the follow-up period, and last visit from these indices were evaluated by using the latent Markov model (LMM). The mean (standard deviation) age of subjects was 44.0 (6.8) years and 73.6% of them were female. LMM identified three latent states of subjects in terms of change in all anthropometric indices: a low, moderate, and high tendency to progress diabetes with the state sizes (29%, 45%, and 26%), respectively. LMM showed that the probability of transitioning from a low to a moderate tendency to progress diabetes was higher than the other transition probabilities. Based on a long-term evaluation of patterns of changes in obesity indices, our results reemphasized the values of all five obesity indices in clinical settings for identifying high-risk prediabetic subjects for developing diabetes in future and the need for more effective obesity prevention strategies.

## Introduction

Type 2 diabetes mellitus (T2DM) is a worldwide public health problem with major morbidities and mortality rate^1^. It is estimated that the number of people with T2DM worldwide will 592 million by 2035^2^. The World Health Organization (WHO) estimated for Iran, there will 5.2 million Iranians with diabetes mellitus by 2025^3^. Subjects before the onset of T2DM are in prediabetes (PD) state in which the subject’s plasma glucose is higher than normal level, but it is not high enough to be diagnosed as diabetes^4^. In recent years, PD prevalence has increased, especially in developing countries. Prediabetic subjects are at a 3–12 times higher risk for developing diabetes compared to the general people^5^. It is estimated that 5–10% of subjects with PD, will develop T2DM in each year^6,7^.

Obesity is a major concern as it is strongly related to the risk of developing diabetes^8,9^. Of the main obesity indices; hip circumference (HC), waist circumference (WC), waist to hip ratio (WHR), and waist to height ratio (WHtR) have been used as measures of abdominal obesity and body mass index (BMI) has been used as a measure of general obesity.

Previous evidences suggested that abnormality in obesity indices is associated with the risk of developing T2DM and PD^10–12^. For instance, a meta-analysis based on the 17 prospective and 35 cross-sectional studies showed that higher BMI, WC, WHR and WHtR associated with the progress of diabetes^13^. Another meta-analysis prospective indicated that higher WHtR and WC were more strongly associated with the development of diabetes. This meta-analysis showed that WHtR did not have a stronger association with risk of incident diabetes than WC^14^. In addition, in a case and control study involving Chinese adults found that higher WC and WHtR had association with increase in the risk of diabetes. Results of this study also showed that WC was positively associated with risk of PD^15^.

Although the association among obesity indices and T2DM has been investigated in various populations, few studies have been conducted to evaluate such association in prediabetic subjects as high risk population^11,13,16^.

Epidemiological studies indicate over a period of 3–5 years, an average of 25% of subjects with PD progress to T2DM, therefore it is crucial to establish appropriate prevention strategies in PD^17^. One’s anthropometric indices is not necessarily stable; especially in PD. Accordingly, it is necessary to apply an appropriate analytical technique that can provide a comprehensive evaluation of diabetic pathophysiology based on changes in their anthropometric measures over time. Therefore, in this study, an advanced statistical method [i.e. latent Markov model (LMM)] was used for tracking the patterns of changes in obesity indices of prediabetic subjects comprehensively.

Previous studies have described the association of BMI, HC, WC, WHR, and WHtR with the risk of diabetes separately and did not evaluate the changes in these indices over time combinatorically^15,18–21^. The LMM, a latent state-switching method, offers a straightforward approach to classify subjects (latent state) according to patterns of change in BMI, HC, WC, WHR, and WHtR over time simultaneously. This method can be used to classify subjects based on changes in the studied variables; within each latent state, people are highly similar to each other and very different from those in other states. On the other hand, these extracted latent states explain the levels of risk for the onset of diabetes in the future. The LMM estimates the probability of moving among states or remaining in the same state. Subjects are assigned to the latent states for which they had the highest probability of belonging to.

Few studies worldwide have been done among first degree relatives (DFR) of type 2 diabetic patients. These subjects are at high risk of affecting by type 2 diabetes in future. On the other hand, people with impaired glucose tolerance or prediabetic patients have a considerable chance of developing diabetes over the future time horizon. These patients can reduce the future risk of type 2 diabetes by changing their lifestyle and adjusting anthropometric indices as important and key risk factors. Strong family history along with impaired glucose tolerance entail more focus on prediabetics FDR for better understanding the behaviours of variables that lead the increased incidence of diabetes in these high risk people. Considering the above-mentioned theoretical capability of LMM, we used this model to evaluate the patterns of changes in all obesity indices combinatorically over time in FDR prediabetic subjects. This was done to identify latent status or to classify these subjects based on the observed changes in obesity measures to indicate which patients are at high risk of developing diabetes in future.

## Materials and methods

### Participants and study design

The current study was conducted under the framework of the Isfahan Diabetes Prevention Study (IDPS). The IDPS was initiated in 2003 among 3483 first-degree relatives (FDRs) from a consecutive sample of patients with T2DM. The IDPS is an ongoing longitudinal study carried out within a cohort of the FDRs of patients with T2DM in Isfahan, which is the largest city in central Iran. The IDPS was implemented to assess the various potential risk factors for diabetes in subjects with a family history of T2DM. The sample of FDR was recruited between 2003 and 2018 and followed up on until 2019. Recruitment methods and examination procedures have been described elsewhere^22^. Subjects with T2DM and normal conditions at the baseline were excluded.

For all participants, was carried out biochemical tests including standard 75 g 2 h oral glucose tolerance test (OGTT), fasting plasma glucose (FPG), and plasma glucose. The participants also completed a self-administered questionnaire that includes information about their health status and various risk factors for diabetes. Of the 3483 FDRs at the baseline, 1228 had been diagnosed with PD.

In the present study, we used data from 1228 prediabetics. The data for those who had at least two measures related to obesity and other laboratory indices during various visits within the follow-up period were used. We used their baseline measurements at their entrance into the cohort, the last measurement, and the mean values of the measurements during the follow-up period for preparing a longitudinal data structure with the least missing data. Therefore, we used three measurements for data analysis in the LMM. Written informed consent was obtained from all subjects in IDPs. The current secondary study has been approved by the Bioethics Committee of Isfahan University of Medical Sciences (IR.MUI.MED.REC.1398.691).

### Anthropometric assessments

At baseline, anthropometric indices were recorded while participants were without footwear and minimally clothed. Weight was measured by a balanced scale and recorded to the nearest 0.1 kg. While subjects were in a normal standing position height was determined using a wall-fixed tape measure and recorded to the nearest 0.5 cm. BMI was calculated by dividing weight (kg) by the square of height (m^2^). WC and HC were determined using a metal tape measure without imposing any pressure on the body surface and were recorded to the nearest 0.5 cm. The location for measuring WC was considered as the narrowest level between the lowest rib and iliac crest, whilst hip circumference was conserved as the largest level. WHR was calculated as dividing WC by HC. The WHtR was calculated as the ratio of waist-to-height.

### Laboratory parameters

Biochemical tests including FPG, and standard 75 g OGTT; at baseline, 30, 60, and 120 min were carried out for all subjects. Post-prandial plasma glucose was measured using venous blood samples at 30 and 60 min after oral glucose administration.

Plasma glucose and lipid profile concentrations were determined using enzymatic colorimetric method (ParsAzmoon, Tehran, Iran) adapted to a Selectra-2 auto-analyzer (Vital Scientific, Spankeren, Netherlands). To determine the lipid profile and FPG, a blood sample was drawn from all subjects after 10–12 h of overnight fasting.

The serum concentration of low-density lipoprotein cholesterol (LDL) was calculated using the Friedwald equation for subjects with serum triglycerides (TG) levels < 400 mg/dL^23^. Serum concentration of high-density lipoprotein cholesterol (HDL), CHOL, and TG were measured using standard procedures^23^.

Definitions and diagnostic criteria were based on the American Diabetes Association (ADA) guidelines. Newly diagnosed diabetes (NDD) was defined as having 2 h PG ≥ 11.1 mmol/L during OGTT or FPG levels ≥ 7.0 mmol/L. PD was defined as having FPG levels between 5.6 and 6.9 mmol/L (IFG), a 2 h PG concentration between 7.8 and 11.0 mmol/L (IGT). Normal subjects were reported as having FPG levels < 5.5 mmol/L^24^. Also, all subjects developing IFG and IGT were pooled in a unique “impaired glucose metabolism” (IGM) group for the analyses.

### Other variables

The subjects completed a demographic questionnaire that included information about their age, gender, marital status, educational level, and smoking status. Physical activity was recorded using an short form of International Physical Activity Questionnaire (IPAQ)^25^. Diastolic blood pressure (DBP) and systolic blood pressure (SBP) were recorded. Blood pressure was measured two times (with at least 30 s intervals between measurements) using a mercury sphygmomanometer while subjects were in a seated position. The mean of two measurements was recorded as the subject’s blood pressure. According to the Joint National Committee (JNC) on the Prevention, Detection, Evaluation, and Treatment of High Blood Pressure and WHO guidelines, hypertension was defined as DBP ≥ 85 mmHg and SBP ≥ 130 mmHg^26^. The questionnaires were administered and collected at the Endocrine and Metabolism Research Center, Isfahan University of Medical Sciences.

### Statistical analysis

Continuous and categorical basic characteristics of the subjects were presented as mean [standard deviation (SD)] and frequency (percentage) and compared between study groups using analysis of variance (ANOVA) or independent samples *t* test and Chi-square tests, respectively.

Three measures from each anthropometric measure were obtained for each study subject and were used to evaluate the pattern of changes in these measures by using LMM^27^.

The process of LMM fitting in the current study was as follows: LMMs with 2-State 1-Class, 2-State 2-Class, 2-State 3-Class, 3-State 1-Class, 3-State 2-Class, and 3-State 3-Class were fitted to data sequentially. Latent classes are unobservable (latent) subgroups or segments comprising people with similar response patterns (in current study, anthropometric indices) irrespective of changes over time in response variables. Subjects are assigned to latent classes based on their posterior class membership probabilities. During LMM fitting subjects can be classified into homogenous subgroups (i.e., latent classes) based on observed response variables and within each extracted latent class subjects differing in their patterns of changes in observed responses over time were grouped into the latent states, which can be conceptualized as indicating subpopulation with independent structures in terms of response variable. In extracted latent states each subject may move between latent states during the time follow up.

LMMs were fitted for increasing values of the number of latent states until the log-likelihood, BIC, and AIC indices decreased with respect to the previous value. The log-likelihood, AIC, and BIC of a model indicate the fit of the model to the data, with a lower value indicating a better fit and parsimony of different models. The number of parameters indicates the parsimony of the model. In order to select the best model we also relied on classification error and entropy indices across different fitted LM models. Lower classification error and higher entropy indicate better model fitting and better state separation^28,29^.

Finally, during the above-mentioned process a model with 3-State 1-Class was selected based on the goodness of fit criteria and higher interpretability. Three latent states were extracted based on patterns of changes in BMI, HC, WC, WHR, and WHtR combinatorically in prediabetic subjects. These states represented different levels of future diabetes progression and were labeled as “State1”, “State2”, and “State3”.

After finalizing the appropriate number of latent states i.e., LMMs without covariate, a LMM with covariates including age, marital status, educational levels, smoking status, physical activity, gender, SBP, DBP, and lipid profile (CHOL, TG, HDL and LDL) was also fitted. The fitted models were adopted separately in gender subgroups. The extracted latent states were interpreted based on the mean values of BMI, HC, WC, WHR, and WHtR.

Initial probabilities for each latent state and transition probabilities for moving between latent states are also estimated. The initial probabilities are defined as the probability of the current state is that the one needed to predict the future. The transition probability is the probability of a subject moving between different latent states. The subjects in any given state can remain or move to other latent states.

The LMMs were fitted using the LMest package^30^ developed within the R free statistical Software (version 3.6.3)^31^. Other statistical analyses were performed using the SPSS software (version 16; SPSS Inc, Chicago, IL, USA).

### Ethics approval and consent to participate

All participants were informed about the study and informed consent was obtained from all the participants. The study adhered to the Declaration of Helsinki and ethics approval was obtained from the Bioethics Committee of Isfahan University of Medical Sciences.

## Results

The mean (standard deviation) age of the 1228 study subjects was 44.0 (6.8) years and 73.6% were female. The mean (SD) of follow up period for current study PD participant was 7.7 (3.81) with median 7 and minimum 1 and maximum 16 years. The prevalence of PD status was statistically significantly different between male and female groups, in which IGT and IGM statues more prevalent among females than males (*P* < 0.001). Mean value of WC was significantly higher in males while mean values of CHOL and HDL were higher in females (*P* < 0.001). The general characteristics of subjects at the baseline across different categories of PD are presented in Table 1. Mean values of BMI, HC, WC and WHR were statistically significantly higher in IGM group while mean value of HDL was higher in IGT group (*P* < 0.001) (Table 1).

**Table 1:** Basic demographic and clinical characteristics of different categories of subjects at the baseline.

Variables	Prediabetic			*P* value
	IFG (n = 560)	IGT (n = 279)	IGM (n = 389)	
Age (years)	43.8 ± 6.7	43.4 ± 7.1	44.6 ± 6.8	0.06*
(Male/female) (%)	(204/356) (36.4/63.6)	(48/231) (17.2/82.8)	(72/317) (18.5/81.5)	< 0.001**
Smoking	29 (5.2)	5 (1.8)	9 (2.3)	0.05**
**Educational levels**				
Illiterate	34 (6.1)	15 (5)	20 (5.1)	0.02**
University graduate	245 (43.8)	150 (53.8)	219 (56.3)	
12 year (diploma)	179 (32)	76 (27.2)	97 (24.9)	
< 12 year	83 (14.8)	37 (13.3)	46 (11.8)	
**Obesity indices**				
BMI (kg/m^2^)	29.3 ± 4.2	29.1 ± 3.8	30.2 ± 4.5	< 0.001*
HC (cm)	107.9 ± 8.7	107.3 ± 8.7	109.1 ± 9.9	0.03*
WC (cm)	91.5 ± 9.6	89.2 ± 9.1	91.7 ± 9.5	< 0.001*
WHR (cm)	0.57 ± 0.06	0.57 ± 0.06	0.58 ± 0.06	< 0.001*
WHtR (cm)	0.85 ± 0.07	0.83 ± 0.06	0.84 ± 0.07	< 0.004*
SBP (mmHg)	11.8 ± 1.4	11.4 ± 1.2	11.7 ± 1.4	0.03*
DBP (mmHg)	7.7 ± 0.96	7.5 ± 0.88	7.7 ± 0.91	0.02*
**Lipid profile**				
CHOL (mg/dl)	201.9 ± 34.8	194.9 ± 33.4	196.3 ± 33.34	0.08*
TG (mg/dl)	169.5 ± 82.6	165.8 ± 90.7	158.0 ± 79.7	0.29*
HDL (mg/dl)	45.4 ± 10.24	46.4 ± 10.7	43.2 ± 10.1	< 0.003*
LDL (mg/dl)	122.2 ± 26.1	116.5 ± 26.2	122.0 ± 27.1	0.06*
Physical activity (min/week)	65.1 ± 79.7	61.4 ± 80.5	23.7 ± 67.4	< 0.001*
FPG (mmol/l)	103.5 ± 8.5	96.6 ± 8.8	103.8 ± 9.9	< 0.001*

Values are mean ± *SD* for continuous and frequency (%) for categorical variables.

*IGT* impaired glucose tolerance, *IFG* impaired fasting glucose, *IGM* impaired glucose metabolism; including subjects with IGT and/or IFG, *BMI* Body mass index, *HC* hip circumference, *WC* waist circumference, *WHR* waist to hip ratio, *WHtR* waist to height ratio, *SBP* systolic blood pressure, *DBP* diastolic blood pressure, *CHOL* total cholesterol, *TG* triglycerides, *HDL* high-density lipoprotein cholesterol, *LDL* low-density lipoprotein cholesterol, *FPG* fasting plasma glucose.

*ANOVA test, **Chi-square test, *P* < 0.05 is considered as significant.

The general characteristics of subjects at the end of follow-up are presented in Table 2. In PD status, mean values of BMI, HC were statistically significantly higher in IFG group than others two PD groups (*P* < 0.001), as well as compared with diabetic group (DM). The mean of FPG in DM group was higher than IFG, IGT, and NGT groups (*P* < 0.001) (Table 2). Over the 16-year follow-up, 339 (27.6%) became diabetic, 204 (16.6%) returned to a normal glycemic status, and 403 (32.8%) remained PD (IGM), respectively. Data regarding the final status of 282 (23%) subjects were not available.

**Table 2:** Basic demographic and clinical characteristics of different categories of subjects at the end of follow-up.

Variables	Prediabetic		NGT (n = 204)	DM (n = 339)	*P *value
	IFG (n = 303)	IGT (n = 100)			
Age (years)	43.9 ± 7.1	42.1 ± 5.2	42.9 ± 6.3	44.5 ± 6.8	< 0.001*
(Male/female) (%)	(66/237) (21.8/78.2)	(23/77) (23/77)	(68/136) (33.3/66.7)	(90/249) (26.5/73.5)	0.03**
Smoking	6 (2)	5 (5)	7 (3.4)	12 (3.5)	0.28**
**Educational levels**					
Illiterate	15 (5)	3 (3)	9 (4.4)	16 (4.7)	0.94**
University graduate	143 (47.2)	53 (53)	101 (49.5)	180 (53.1)	
12 year (diploma)	95 (31.4)	28 (28)	58 (28.4)	97 (28.6)	
< 12 year	42 (13.9)	15 (15)	31 (15.2)	41 (12.1)	
**Obesity indices**					
BMI (kg/m^2^)	30.0 ± 4.3	29.7 ± 3.8	28.9 ± 3.9	30.5 ± 4.5	< 0.001*
HC (cm)	106.2 ± 8.7	104.7 ± 6.9	104.6 ± 8.0	108.1 ± 9.5	< 0.001*
WC (cm)	94.9810.1	94.5 ± 9.9	93.9 ± 10.3	95.5 ± 9.9	0.31*
WHR (cm)	0.60 ± 0.60	0.60 ± 0.06	0.58 ± 0.06	0.60 ± 0.06	0.04*
WHtR (cm)	0.89 ± 0.07	0.90 ± 0.08	0.90 ± 0.08	0.88 ± 0.07	0.08*
SBP (mmHg)	11.6 ± 1.4	11.6 ± 1.3	11.6 ± 1.3	11.7 ± 1.4	0.78*
DBP (mmHg)	7.6 ± 0.85	7.7 ± 0.9	7.7 ± 0.93	7.7 ± 0.97	0.97*
**Lipid profile**					
CHOL (mg/dL)	198.3 ± 36.3	196.9 ± 33.7	194.6 ± 29.8	199.7 ± 34.5	0.51**
TG (mg/dL)	159.5 ± 84.4	159.4 ± 79.2	147.9 ± 73.3	181.7 ± 89.4	< 0.001*
HDL (mg/dL)	45.2 ± 10.03	44.9 ± 11.2	45.7 ± 9.7	43.7 ± 10.8	0.25*
LDL (mg/dL)	122.4 ± 26.5	120.6 ± 28.7	118.2 ± 24.6	120.5 ± 27.5	0.54*
Physical activity (min/week)	88.6 ± 91.7	106.8 ± 88.8	91.0 ± 87.8	79.7 ± 74.9	0.72*
FPG (mmol/l)	103.7 ± 8.2	96.7 ± 7.9	96.8 ± 7.9	105.1 ± 10.1	< 0.001*

Values are mean ± *SD* for continuous and frequency (%) for categorical variables.

*IGT* impaired glucose tolerance, *IFG* impaired fasting glucose, *NGT* normal glucose tolerance, *DM* diabetes group, *BMI* Body mass index, *HC* hip circumference, *WC* waist circumference, *WHR* waist to hip ratio, *WHtR* waist to height ratio, *SBP* systolic blood pressure, *DBP* diastolic blood pressure, *CHOL* total cholesterol, *TG* triglycerides, *HDL* high-density lipoprotein cholesterol, *LDL* low-density lipoprotein cholesterol, *FPG* fasting plasma glucose.

*ANOVA test, **Chi-square test, *P* < 0.05 is considered as significant.

**Figure 1:**
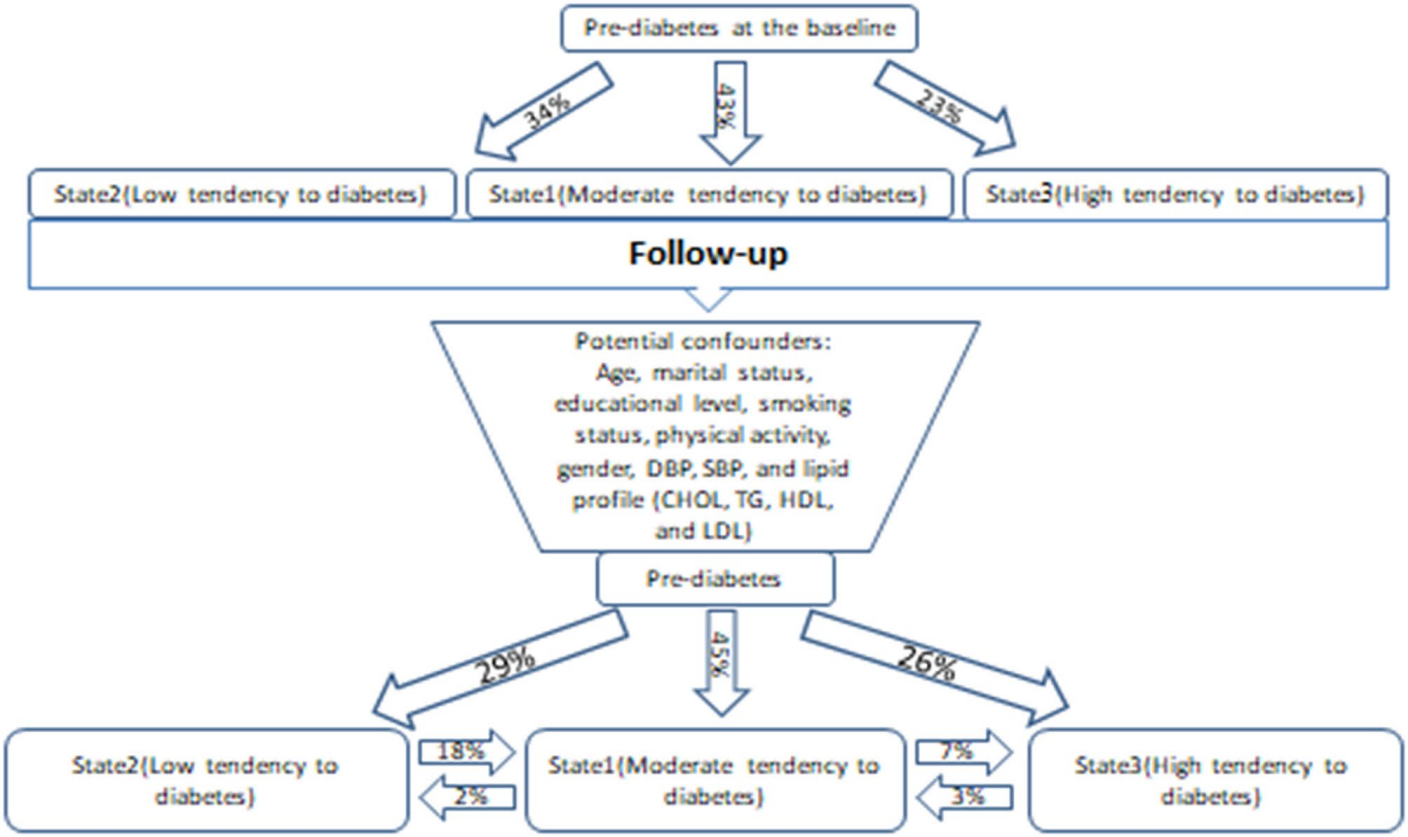
Overview of estimated latent sates sizes and transition probabilities of moving from one state to other state based on the patterns of changes in obesity indices in total sample (Drawn in Microsoft PowerPoint 2010, https://www.microsoft.com/en-us/download/confirmation.aspx?id=20873) created by Shahla Safari (Email: shahlasafari290@gmail.com).

Table 3 presents the results of fitting the LMM regarding the identified latent states of subjects based on BMI, HC, WC, WHR, and WHtR on the total sample, as well as for male and female samples. Three latent states were identified for the total, male, and female samples. The latent states were interpreted based on the means changes of BMI, HC, WC, WHR, and WHtR. State1 consists of subjects with moderate mean values of BMI, HC, WC, WHR, and WHtR. Accordingly, the subjects contained in this state were at a moderate risk of diabetes progression in the future. State2 consists of subjects who had lower mean values of BMI, HC, WC, WHR, and WHtR during the follow-up period. Hence, the subjects in this state are considered as prediabetic patients with a lower tendency of diabetes progression in the future. State3 consists of subjects who had higher mean values of BMI, HC, WC, WHR, and WHtR during the follow up period. Hence, the subjects in this state are considered as having a higher tendency of diabetes progression in the future. The estimated latent state sizes for the total sample based on evaluations of changes in BMI, HC, WC, WHR, and WHtR for State1, State2, and State3, are 45%, 29%, and 26%, respectively (Fig. 1). The sizes of extracted latent states reflect the proportions of subjects whose diabetes tended to progress throughout the follow-up period. The size of extracted latent states based on all obesity indices for female and male participants are also presented in Table 3. As can be seen, similar features in terms of latent states structure and size occurred in males and females, in both LMMs with and without adjustment for potential confounders.

**Table 3:** The identified latent states of prediabetic subject resulted from latent Markov analysis.

Group	Obesity indices	Levels of diabetes tendency (without covariates)			Levels of diabetes tendency (with covariates)		
		Moderate (State1)	Low (State2)	High (State3)	Moderate (State1)	Low (State2)	High (State3)
Total (*n* = 1228)	State size	0.45	0.29	0.26	0.45	0.29	0.26
	Mean of BMI (kg/m^2^)	29.37	25.48	34.98	29.38	25.44	34.98
	Mean of WC (cm)	106.33	99.55	117.58	106.36	99.42	117.53
	Mean of HC (cm)	93.68	82.41	102.87	93.61	82.45	102.48
	Mean of WHR	0.88	0.83	0.87	0.88	0.83	0.88
	Mean of WHtR	0.58	0.52	0.65	0.58	0.52	0.65
Males (*n* = 324)	State size	0.45	0.31	0.24	0.45	0.32	0.23
	Mean of BMI (kg/m^2^)	28.41	25.22	32.57	28.45	25.26	32.26
	Mean of WC (cm)	104.59	99.35	111.87	0.94	0.89	0.97
	Mean of HC (cm)	98.67	88.78	108.04	98.77	88.87	108.13
	Mean of WHR	0.94	0.89	0.97	0.94	0.89	0.97
	Mean of WHtR	0.58	0.52	0.64	0.58	0.52	0.64
Females (*n* = 904)	State size	0.43	0.31	0.26	0.43	0.32	0.25
	Mean of BMI (kg/m^2^)	30.09	25.63	35.65	30.12	25.66	35.70
	Mean of WC (cm)	107.96	99.77	118.56	108.01	99.84	118.64
	Mean of HC (cm)	91.31	81.12	102.52	91.40	81.16	102.61
	Mean of WHR	0.85	0.81	0.87	0.85	0.81	0.87
	Mean of WHtR	0.58	0.52	0.66	0.59	0.52	0.66

The covariates include: age, gender, marital status, educational level, smoking, physical activity, SBP, DBP, and lipid profile (CHOL, TG, HDL, and LDL).

Table 4 presents the estimated initial and transition probabilities for each latent state and for moving from one state to the other states. The estimated initial probabilities (showed under State0) based on all obesity indices ranged from 0.23 to 0.43, indicating that a high proportion of study participants are in State1, which is a moderate-risk state than State2 and State3 in terms of diabetes progression. Other data presented in Table 4 are related to transition probabilities. These transition probabilities have been estimated based on all obesity indices simultaneously. For the total sample, in the adjusted model for confounders, for latent State1 (moderate-risk for diabetes progression) the probabilities of moving to the second latent state (low-risk of diabetes progression) and the third latent state (high-risk of diabetes progression) are 0.02 and 0.07, respectively. For State2, the probability to move to the first latent state is 0.18 (Fig. 1). Also, the probabilities for retaining at the same states are 0.91, 0.82, and 0.97 for State1, State2, and State3, respectively. Similar results were observed when we estimated transition probabilities for male and female samples separately. These transition probabilities indicate that it is not likely that those in State2 will move to State3 and vice versa.

**Table 4:** Initial and transition's probabilities of prediabetic subjects resulted from latent Markov analysis.

Group	Latent status	Levels of diabetes tendency (without covariates)			Levels of diabetes tendency (with covariates)		
		Moderate (State1)	Low (State2)	High (State3)	Moderate (State1)	Low (State2)	High (State3)
Total (*n* = 1228)	State0	0.43	0.34	0.23	0.43	0.33	0.24
	State1	0.91	0.02	0.07	0.91	0.02	0.07
	State2	0.18	0.82	0.00	0.18	0.82	0.00
	State3	0.03	0.00	0.97	0.03	0.01	0.96
Males (*n* = 324)	State0	0.46	0.35	0.19	0.45	0.35	0.19
	State1	0.86	0.04	0.10	0.86	0.04	0.10
	State2	0.15	0.84	0.01	0.15	0.84	0.01
	State3	0.04	0.01	0.95	0.05	0.01	0.94
Females (*n* = 904)	State0	0.42	0.37	0.21	0.42	0.37	0.21
	State1	0.87	0.03	0.10	0.86	0.03	0.11
	State2	0.20	0.80	0.00	0.21	0.79	0.00
	State3	0.02	0.00	0.98	0.02	0.00	0.98

The state0 is initial state. Probabilities represent the probability of transition from a particular state to other states from row to column.

## Discussion

In the present prospective study, which was conducted under the framework of an ongoing cohort study, we followed 1228 prediabetic subjects from 2003 to 2019. Changes in the BMI, HC, WC, WHR, and WHtR were evaluated by using LMM over time. Three latent states were extracted based on the patterns of changes in the mean values of anthropometric indices. The latent states were characterised according to the tendency for affecting by diabetes in the future (low/moderate/high) and had latent state sizes of (29%/45%/26%).

The current study is the first one that classified prediabetic subjects into homogeneous subgroups based on the changes in mean values of BMI, HC, WC, WHR, and WHtR over time by using an advanced statistical model. However, there are some studies that have investigated the general population, as well as some specific populations, by applying simple statistical approaches^16,19^. In these studies, the association between BMI, WC, HC, WHR, and WHtR with the risk of developing diabetes in the future has been considered separately. For instance, Sayeed et al. showed a significant association between WHtR and risk diabetes progression^11^. In a meta-analysis based on the individual data of the Asian cohorts, Qiao et al. showed that in all studies included in this review, either BMI, WC, and WHR predicted or was associated with T2DM, separately^13^.

Obesity indices abnormality is a strong risk factor for T2DM^16,20,32^. In the present study, the subjects with a high tendency for diabetes progression had obesity indices abnormalities. We found that the mean of obesity indices was proportionally associated with a low, moderate, and high tendency for diabetes progression. Our results were in accordance with the results of previous studies that have focused on the association of obesity disorders with the risk of diabetes progression^18,19^.

Several studies have indicated an association of BMI with T2DM in prediabetic subjects^18,33,34^. The results of the present study are consistent with previous studies in which the subjects with a high tendency for diabetes progression had proportionally a higher BMI mean value. Wei et al. obtained similar results to our study in terms of the association of BMI with future diabetes risk^34^. In another study, Shakeri et al. found a relationship between anthropometric indices and diabetes. They reported that the odds ratio of affecting by diabetes can elevate with increasing BMI^33^. Furthermore, Haghighatdoost et al.'s study suggested that BMI is strongly associated with diabetes incidence and WC was moderately related to diabetes incident^20^. Denmark et al.’s study indicated that those who were overweight were more likely to develop diabetes (1.1% per unit of increase in the BMI). The study also revealed that the risk of diabetes increases with weight gain and obesity^35^.

WC and WHR are associated with the progression of T2DM^8,36^. In evaluating the simultaneous association of WC and WHR with T2DM, our findings implied the subjects with a high tendency of diabetes progression had a higher mean WC and WHR than other subjects. Klein et al. showed WC is a simple measure that can be used to identify subjects at increased risk of T2DM^36^. In line with the present study, Sargeant et al. demonstrated WHR significantly associated with diabetes progression in future^8^.

It has previously been demonstrated by some studies that WHtR is a predictor of T2DM in prediabetic subjects^32,37^. In the present study, the subjects with a high tendency for diabetes progression had a higher mean value of WHtR. In line with the present study, Tulloch-Reid et al. showed a strong association between BMI and WHtR abnormality with T2DM^37^. In a longitudinal study, Hadaegh et al. showed WHtR yielded the highest ability for the future development of diabetes between other anthropometric measures^32^. In the present study, the subjects with a high tendency of diabetes progression had a higher mean value of obesity indices. This finding is in line with the results of Nayak et al., a study that has emphasised the association WC, HC, and BMI with the future risk of diabetes in subjects with PD^38^.

Most studies have ignored the complex, unstable, and variable conditions of prediabetic people in terms of obesity indices. Yearly, 5–10% of people with PD will progress to diabetes, with the same proportion converting back to normoglycemia^39^. In contrast to other studies, in the current survey, we followed and evaluated the trajectories of five important obesity indices simultaneously over a long-time horizon and identified people with a low, moderate, and high probability to progress diabetes or to remain in the same condition in the future. The results showed that the probability of transitioning from having a moderate tendency to develop diabetes to having a low tendency to develop diabetes was lower than the probability of transitioning in the opposite direction. Also, the probability of staying in the same state was higher than that of transitioning to different states.

Based on a long-term evaluation of changes in general and abdominal obesity indices, we classified PD subjects as being at either a low, moderate and high risk for future diabetes progression. Also, the method used enabled us to estimate the transition probabilities from low- to moderate and to high-risk states and vice versa. In conclusion, our results reemphasised the relevance of all five obesity measures in clinical settings for identifying prediabetic subjects with a high risk of diabetes progression. Our results also indicate the need to quickly conduct effective prevention strategies, in the area of controlling obesity, for prediabetics who are at a high risk of becoming diabetic.

## Data Availability

The data that support the findings of this study are available from the corresponding author upon reasonable request.
